# Obesity Enhances the Conversion of Adipose-Derived Stromal/Stem Cells into Carcinoma-Associated Fibroblast Leading to Cancer Cell Proliferation and Progression to an Invasive Phenotype

**DOI:** 10.1155/2017/9216502

**Published:** 2017-12-17

**Authors:** Amy L. Strong, Dorothy T. Pei, Christian G. Hurst, Jeffrey M. Gimble, Matthew E. Burow, Bruce A. Bunnell

**Affiliations:** ^1^Center for Stem Cell Research and Regenerative Medicine, Tulane University School of Medicine, New Orleans, LA, USA; ^2^LaCell LLC, New Orleans, LA 70112, USA; ^3^Departments of Medicine and Surgery, Tulane University School of Medicine, New Orleans, LA 70112, USA; ^4^Department of Medicine, Section of Hematology and Medical Oncology, Tulane Health Sciences Center, New Orleans, LA 70112, USA

## Abstract

Obesity is associated with enhanced tumor growth and progression. Within the adipose tissue are adipose-derived stromal/stem cells (ASCs) that have been shown to convert into carcinoma-associated fibroblast (CAFs) in the presence of tumor-derived factors. However, the impact of obesity on the ASCs and on the conversion of ASCs into CAFs has not been demonstrated. In the current study, ASCs isolated from lean donors (BMI < 25; lnASCs) were compared with ASCs isolated from obese donors (BMI > 30, obASCs). The contribution of tumor-derived factors on the conversion of ASCs to CAFs was investigated. Following exposure to cancer cells, obASCs expressed higher levels of CAF markers, including NG2, alpha-SMA, VEGF, FAP, and FSP, compared to lnASCs. To investigate the crosstalk between ASCs and breast cancer cells, MCF7 cells were serially cocultured with lnASCs or obASCs. After coculture with lnASCs and obASCs, MCF7 cells demonstrated enhanced proliferation and expressed an invasive phenotype morphologically, with more pronounced effects following exposure to obASCs. Long-term exposure to obASCs also enhanced the expression of protumorgenic factors. Together, these results suggest that obesity alters ASCs to favor their rapid conversion into CAFs, which in turn enhances the proliferative rate, the phenotype, and gene expression profile of breast cancer cells.

## 1. Introduction

Adipose-derived stem/stromal cells (ASCs) are multipotent stromal cells isolated from adipose tissue and have been used for a wide variety of tissue engineering applications. Their multipotency, immunomodulatory properties, and regenerative potential have made ASCs an attractive candidate for clinical applications. However, studies have also shown the paradoxical effect of ASCs in promoting cancer [1, 2]. Numerous studies have shown that soluble factors secreted by cancer cells reprogram ASCs to secrete growth factors, cytokines, and ECM-remodeling proteins, converting these cells into carcinoma-associated fibroblast- (CAF-) like cells [3–6]. CAFs display traits of myofibroblast and are abundant in the most invasive human breast cancers [7]. It has been shown that CAFs stimulate tumor growth and promote angiogenesis through the secretion of growth factors and proinflammatory cytokines, such as interleukins and interferons [8, 9]. Moreover, CAFs alter the malignant potential of cancer cells by promoting the secretion of proinvasive factors, such as matrix metalloproteinases. Lastly, CAFs have been shown to alter the extracellular matrix of breast and adipose tissue. Differentiation of ASCs into CAFs results in the expression alpha-smooth muscle actin (*α*-SMA), fibroblast activation protein alpha (FAP1), and fibroblast-specific protein (FSP) in addition to the production of ECM proteins, such as collagens and fibronectin, which enhances the stiffness of cells [10]. Increased stiffness has been shown to promote the proliferation and metastasis of breast cancer cells [11, 12]. Collectively, these studies have shown that ASCs may differentiate into CAFs and increase tumorigenesis, angiogenesis, cancer cell invasion and metastasis, resistance to chemotherapy, and cancer recurrence [13–19].

Obesity has reached epidemic levels in recent years and has become a major risk factor for breast cancer development [20, 21]. In particular, studies have shown that a high body mass index (BMI) at disease onset has predictive value for poor prognosis in breast cancer patients [20, 21]. Excessive adipose tissue has been associated with an altered cytokine profile, characterized by a reduction in the release of anti-inflammatory cytokines and an increased secretion of proinflammatory cytokines [22]. Consequently, obesity is considered to be an important source of low-grade chronic inflammation [23]. Prolonged exposure to high levels of proinflammatory cytokines secreted by adipose tissue before tumor formation may provide an environment conducive to the development and subsequent metastasis of breast cancer. Therefore, it is pertinent to determine the effects of obesity on ASC biology, as this may shed light on the precise mechanism(s) by which obesity may increase the morbidity and mortality of breast cancer.

Several studies investigating the effects of obesity on ASC biology have shown changes in the transcriptomic profile and immunomodulatory potential of ASCs isolated from obese subjects [24, 25]. Additional studies have shown that coculture of these ASCs isolated from obese subjects (obASCs) results in increased tumor growth and metastasis of breast cancer cells [26, 27]. While these previous studies have investigated the short-term effects of obASC exposure, few studies have investigated the effects of long-term exposure of obASCs on breast cancer cells and cancer cell proliferation. In the present study, we used serial coculture of obASCs with breast cancer cells to investigate the impacts on the ASCs and breast cancer cells. When compared to ASCs isolated from lean subjects (lnASCs), serial coculture with cancer cells resulted in the rapid and robust expression of CAF markers in obASCs. Analysis of cytokines secreted in the media during coculture revealed higher levels of cytokines secreted by obASCs. Further analysis of cancer cells revealed the rapid proliferation of cancer cells after long-term coculture with obASCs, whereas the effect was less pronounced following exposure to lnASCs. The results suggest that soluble factors produced by cancer cells induce the conversion of obASCs into CAFs more readily than lnASCs, leading to increased secretion of cytokines and chemokines from obASCs that reciprocally support the growth of cancer cells.

## 2. Material and Methods

### 2.1. Human Subjects

Human ASCs were obtained from 12 Caucasian females (2 groups, 6 donors per group) undergoing elective liposuction procedures, as previously described [28]. All protocols were reviewed and approved by the Pennington Biomedical Research Center Institutional Review Board, and all human participants provided written informed consent. Briefly, ASCs were isolated from processed lipoaspirates from the subcutaneous abdominal adipose tissue of lean or obese patients. Liposuction aspirates were incubated in 0.1% type I collagenase (Sigma) and 1% powdered bovine serum albumin (BSA, fraction V; Sigma) dissolved in 100 ml of phosphate buffered saline (PBS) supplemented with 2 mM calcium chloride. The mixture was placed in a 37°C shaking water bath or incubator at 75 rpm for 60 min and then centrifuged to remove oil, fat, primary adipocytes, collagenase solution, and cellular debris. The resulting cell pellet was resuspended in stromal medium (SM), which consisted of Dulbecco's modified eagles medium (DMEM)/F12 (Hyclone, Logan UT), 10% fetal bovine serum (Hyclone, Logan UT), and 1% antibiotic/antimycotic (Thermo Fisher Scientific, Waltham, MA), and plated in 175 cm^2^ flasks. Fresh SM was added every 2-3 days until cells achieved 80–90% confluence, at which time cells were harvested with 0.25% trypsin/1 mM EDTA (GIBCO, Thermo Fisher Scientific) and cryopreserved prior to experimental use. The mean BMI for the lnASC group was 22.7 ± 1.9, while the mean BMI for the obASCs was 32.7 ± 3.7. The mean age of the subjects for each group of donors was as follows: 38.8 ± 7.0 for lnASCs and 42.5 ± 8.9 for obASCs. No statistical significance in age was observed between the donor groups.

### 2.2. Cell Culture

#### 2.2.1. ASCs

Frozen vials of ASCs were thawed and cultured on 150 cm^2^ culture dishes (Nunc, Rochester, NY) in 25 ml complete culture medium (CCM), which consisted of *α*-minimal essential medium (*α*MEM; GIBCO; Grand Island, NY), 20% fetal bovine serum (FBS; Atlanta Biologicals, Lawrenceville, GA), 100 units per ml penicillin/100 *μ*g/ml streptomycin (P/S; GIBCO), and 2 mM L-glutamine (GIBCO). The cells were then incubated at 37°C with 5% humidified CO_2_. After 24 hours, viable cells were harvested with 0.25% trypsin/1 mM EDTA and replated at 100–200 cells/cm^2^ in CCM. The medium was changed every 2-3 days. For all experiments, subconfluent cells (≤70% confluent) between passages 2–6 were used. Characterization of stem cells was previously performed and published [26].

#### 2.2.2. Breast Cancer Cell (BCC) Lines

MCF7 (HTB-22) cells were obtained directly from American Type Culture Collection (ATCC; Manassas, VA) and used for fewer than 6 months after resuscitation. MCF7 cells were chosen for the specific purpose that they represent a luminal-type breast cancer. Cell line authentication was conducted by ATCC via short tandem repeat profiling. Cells were cultured in Dulbecco's modified eagle's medium (DMEM; GIBCO), supplemented with 10% FBS and P/S. Cells were grown at 37°C with 5% humidified CO_2_, fed every 2-3 days, and split 1 : 4 to 1 : 6 when the cells reached 90% confluence. We followed the methods of Strong et al. [27].

### 2.3. Generation of GFP^+^ MCF7 Cells and RFP^+^ ASCs

To produce lentivirus, 293T cells were transfected through a modified calcium chloride transfection protocol when cells reached 70–75% confluence. For each transfection, 10 *μ*g of packaging plasmid, enveloping encoding plasmid, and transfer plasmid containing GFP and neomycin resistance or dsRed and neomycin resistance were used. After 48 hours, the medium was harvested and used to transduce cancer cells. To transduce MCF7 cells, conditioned medium containing virus with GFP and neomycin resistance was added to MCF7 cells at 70% confluence. To transduce ASCs, conditioned medium containing virus with dsRed and neomycin resistance was added to ASCs at 70% confluence. MCF7 cells and ASCs were selected with 500 *μ*g/ml of Geneticin (Invitrogen; Carlsbad, CA) for 2 weeks and GFP expression or dsRed expression was verified with flow cytometry. All MCF7 cells and ASCs used for experimentation expressed GFP and dsRed, respectively. ASCs were characterized based on cell surface marker profile, colony-forming unit assay, and differentiation capacity as shown previously [26].

### 2.4. Proteome Profiler Cytokine Array

MCF7 cells were cultured alone or cocultured with lnASCs or obASCs in DMEM medium containing 10% FBS and P/S. After 7 days, the conditioned media was collected from the three conditions (MCF7, MCF7 with lnASCs, and MCF7 with obASCs) and stored in aliquots at −80°C prior to experimentation. The Proteome Profiler Human XL Cytokine Array (R&D Systems, Minneapolis, MN) was used according to manufacturer's instructions to detect 102 different cytokines directly in the biological replicates. Briefly, the conditioned media was added to cocktail of biotinylated antibodies and incubated at room temperature for 1 hour. The sample antibody mixture was subsequently incubated at 4°C for 19 hours with a membrane embedded with antibodies specific to the cytokines analyzed. Following a washing step, 3 ml of a 1 : 1000 dilution of secondary antibody conjugated with streptavidin-HRP was added to each membrane and incubated at room temperature for 45 minutes. For detection, the membranes were visualized with chemiluminescence reagent provided with the kit on an ImageQuant LAS 4000 (GE Healthcare Life Science; Piscataway, NJ). Quantitative analysis of the protein array was conducted with densitometry.

### 2.5. MCF7 Cell and ASC Serial Coculture

MCF7 cells were cocultured with lnASCs (*n* = 6 donors) or obASCs (*n* = 6 donors) in a 1 : 1 ratio for a total of 100,000 cells in DMEM supplemented with 10% FBS and P/S. After 7 days, cocultured cells were harvested, washed, and FACS sorted with the Becton Dickinson FACSVantage SE Cell Sorter with DiVa option (BD, Franklin Lakes, NJ) based on dsRed expression (ASCs). After one coculture, cells were denoted with c1, for example, cancer cells following the initial coculture would be denoted lnMCF7(c1) or obMCF7(c1). Cells serially cocultured two times (c2) were generated from naïve MCF7 cells cocultured with lnASC(c1) or obASC(c1). After 7 days, these serially cocultured cells were FACS sorted, enriching for lnASC(c2) or obASC(c2). To generate serially cocultured MCF7 cells, naïve lnASCs were cocultured with lnMCF7(c1) and naïve obASCs were cocultured with obMCF7(c1). After 7 days, these serially cocultured cells were sorted into lnMCF7(c2) and obMCF7(c2). Serial cocultures with the cancer cells were conducted until c4. Naïve MCF7 cells, naïve lnASCs, and naïve obASCs without previous coculture were collected and served as controls.

### 2.6. RNA Isolation Followed by Reverse Transcriptase Polymerase Chain Reaction (qRT-PCR)

Serially cocultured and FACS sorted MCF7 cells, lnASCs, or obASCs were analyzed by qRT-PCR. RNA was extracted using TRIzol reagent (Invitrogen), purified with RNeasy columns (Qiagen), and digested with DNase I (Invitrogen). A total of 2 *μ*g of cellular RNA was used for cDNA synthesis with SuperScript VILO cDNA synthesis kit (Invitrogen). Quantitative real-time PCR was performed using the EXPRESS SYBR GreenER qPCR SuperMix Kit (Invitrogen) according to the manufacturer's instructions. Primer sequences used are located in Table 1. At the completion of the reaction, ΔΔCt was calculated to quantify mRNA expression.

### 2.7. Alamar Blue Cell Proliferation Assay

Alamar blue cell proliferation assay was conducted according to the manufacturer's instructions. Briefly, 100 cells were sorted into a 96-well plate in triplicates with FACS CloneCyte device and software (Becton Dickinson). After the cells adhered overnight, the medium was removed, the wells were washed with PBS, and the cells were incubated in 10% Alamar blue reagent (Invitrogen). After overnight culture, the fluorescence intensity was measured at an excitation wavelength of 540 nm and an emission wavelength of 580 nm using a fluorescence plate reader (FLUOstar Optima; BMG Labtech Inc., Durham, NC). Cells were assessed on days 1, 2, 4, and 7.

### 2.8. Morphological Assessment

To assess the morphology of MCF7 cells after continuous exposure to lnASCs or obASCs, FACS sorted MCF7(c1), lnMCF7(c1), obMCF7(c1), MCF7(c4), lnMCF7(c4), and obMCF7(c4) were visualized with Nikon Eclipse TE200 (Melville, NY). Images were acquired on Nikon Digital Camera DXM1200F using the Nikon ACT-1 software version 2.7.

### 2.9. Statistical Analysis

All values are presented as mean ± standard error of the mean (SEM). The statistical differences among three or more groups were determined by ANOVA, followed by post hoc Tukey multiple comparison tests versus the respective control group. Statistical significance was set at *P* < 0.05. The analysis was performed using Prism (GraphPad Software, San Diego, CA).

## 3. Results

### 3.1. Obesity Alters the Secretome Profile of Cocultured Cells

**Figure 1:**
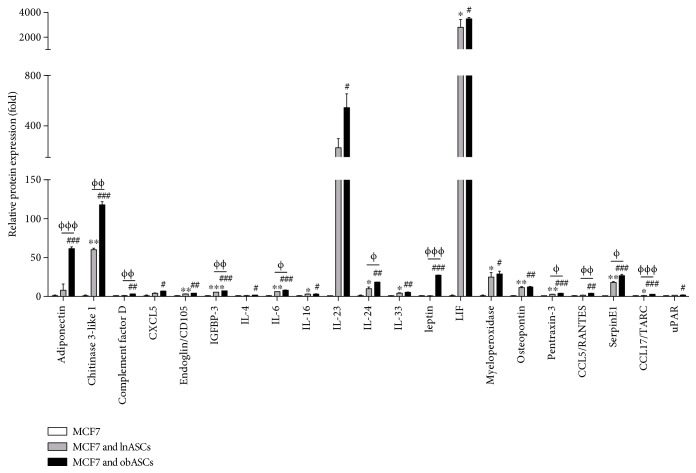
Secretome of MCF7 cells differs from secretome of MCF7 cells cocultured with lnASCs and obASCs. MCF7 cells were cultured alone or cocultured with lnASCs or obASCs for 7 days. The levels of various factors in the supernatants were measured by Proteome Profiler Cytokine Array at day 7 and were normalized to the levels observed in the media of MCF7 cells cultured alone. Bar: ±SEM. ^∗^*P* < 0.05; ^∗∗^*P* < 0.01; ^∗∗∗^*P* < 0.001 between MCF7 and MCF7/lnASCs. ^#^*P* < 0.05; ^##^*P* < 0.01; ^###^*P* < 0.001 between MCF7 and MCF7/obASCs. ^Φ^*P* < 0.05; ^ΦΦ^*P* < 0.01; ^ΦΦΦ^*P* < 0.001 between MCF7/lnASCs and MCF7/obASCs.

The secretome profiles of MCF7 cells cultured alone and cocultured with lnASCs or obASCs were assessed with the proteome profiler array. Of the 102 cytokines assessed, the array showed increased expression of 21 proteins in the cocultured samples: adiponectin, chitinase 3-like 1, complement factor D, CXCL5, endoglin/CD105, IGFBP-3, IL-4, IL-6, IL-16, IL-23, IL-24, IL-33, leptin, LIF, myeloperoxidase, osteopontin, pentraxin-3, CCL5/RANTES, serpinE1, CCL17/TARC, and uPAR. Of these 21 proteins, 11 factors were overexpressed in the MCF7/obASCs compared to the MCF7/lnASCs group: adiponectin (61.5-fold versus 8.0-fold, *P* < 0.001), chitinase 3-like 1 (117.8-fold versus 60.1-fold, *P* < 0.01), complement factor D (3.3-fold versus 1.2-fold, *P* < 0.01), IGFBP-3 (7.3-fold versus 5.6-fold, *P* < 0.01), IL-6 (8.1-fold versus 6.4-fold, *P* < 0.05), IL-24 (18.4-fold versus 10.0-fold, *P* < 0.05), leptin (27.5-fold versus 0.9-fold, *P* < 0.001), pentraxin-3 (4.1-fold versus 2.9-fold, *P* < 0.05), CCL5/RANTES (4.2-fold versus 1.7-fold, *P* < 0.01), serpinE1 (23.8-fold versus 18.1-fold, *P* < 0.05), and CCL17/TARC (3.0-fold versus 1.3-fold, *P* < 0.001) (Figure 1).

### 3.2. Serial MCF7 Coculture Leads to More Robust Expression of CAF Markers in obASCs Compared to lnASCs

**Figure 2:**
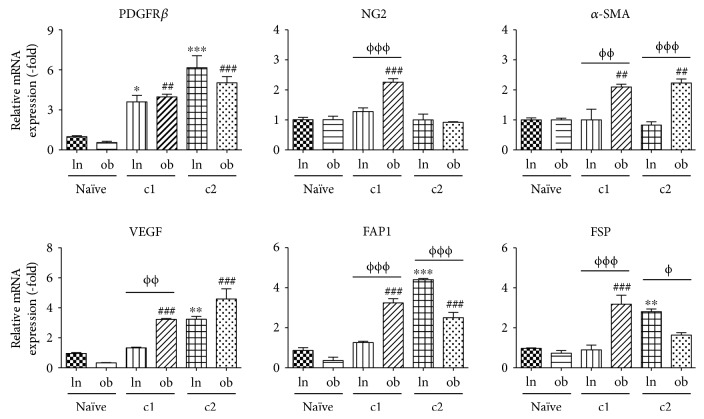
lnASCs and obASCs demonstrated enhanced expression of CAF markers following coculture. lnASCs and obASCs were serially cocultured with MCF7 cells. After 7 days, the cocultured cells (c1) were sorted, and the gene expression of various CAF markers in lnASCs and obASCs was assessed by qRT-PCR. The sorted lnASC(c1) or obASC(c1) cells were cocultured with MCF7 cells for another 7 days and sorted. Expression of CAF markers in lnASCs was assessed by qRT-PCR and normalized to the levels observed in lnASCs without exposure to MCF7 cells (naïve). Bar: ± SEM. ^∗^*P* < 0.05; ^∗∗^*P* < 0.01; ^∗∗∗^*P* < 0.001 compared to naïve lnASCs. ^##^*P* < 0.01; ^###^*P* < 0.001 compared to naïve obASCs. ^Φ^*P* < 0.05; ^ΦΦ^*P* < 0.01; ^ΦΦΦ^*P* < 0.001 between lnASCs and obASCs.

To determine whether the rate of conversion of ASCs to CAFs varies between lnASCs and obASCs following exposure to cancer cells, we investigated the expression of CAF markers in ASCs temporally. Serial coculture with MCF7 cells enhanced the gene expression of PDGFR-beta in lnASCs and obASCs; however, no statistically significant difference was observed between lnASCs and obASCs. In contrast, following coculture with MCF7 cells, the expression of several genes in obASCs was enhanced compared to lnASCs: NG2 (2.3-fold in obASCs and 1.2-fold in lnASCs, *P* < 0.001), *α*-SMA (2.1-fold in obASCs and 1.0-fold in lnASCs, *P* < 0.01), VEGF (3.2-fold in obASCs and 1.3-fold in lnASCs, *P* < 0.01), FAP1 (3.2-fold in obASCs and 1.3-fold in lnASCs, *P* < 0.001), and FSP (3.1-fold in obASCs and 0.9-fold in lnASCs, *P* < 0.001; Figure 2). Following two serial cocultures, obASCs (2.2-fold) continued to demonstrate expression of *α*-SMA compared to lnASCs (0.8-fold, *P* < 0.001; Figure 2). Interestingly, the expressions of FAP1 and FSP in lnASCs were higher than those in obASCs after exposure to MCF7 for two serial cocultures: FAP1 (4.4-fold in lnASCs versus 2.5-fold in obASCs, *P* < 0.001) and FSP (2.8-fold in lnASCs versus 1.6-fold in obASCs, *P* < 0.05; Figure 2).

### 3.3. Serial Coculture with MCF7 Cells Leads to Enhanced Expression of Cytokines in obASCs Compared to lnASCs

**Figure 3:**
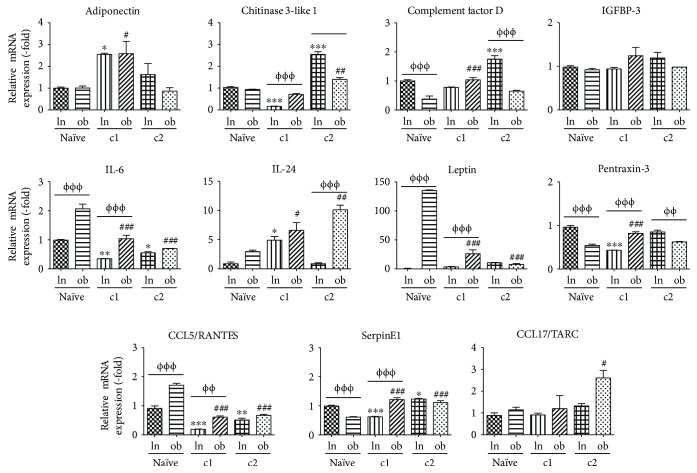
lnASCs and obASCs demonstrated enhanced expression of cytokines following serial coculture. lnASCs and obASCs were serially cocultured with MCF7 cells. After 7 days, the cocultured cells (c1) were sorted, and the gene expression of various factors in lnASCs and obASCs was assessed by qRT-PCR. The sorted lnASC(c1) or obASC(c1) cells were cocultured with MCF7 cells for another 7 days and sorted. The gene expression of various factors in lnASCs was assessed by qRT-PCR and normalized to the levels observed in lnASCs without exposure to MCF7 cells (naïve). Bar: ±SEM. ^∗^*P* < 0.05; ^∗∗^*P* < 0.01; ^∗∗∗^*P* < 0.001 compared to naïve lnASCs. ^#^*P* < 0.05; ^##^*P* < 0.01; ^###^*P* < 0.001 compared to naïve obASCs. ^ΦΦ^*P* < 0.01; ^ΦΦΦ^*P* < 0.001 between lnASCs and obASCs.

Further analysis was conducted in lnASCs and obASCs before coculture to determine if these cells express different levels of cytokines at baseline and to determine whether the stem cells or the cancer cells were secreting the cytokines. The expressions of the following cytokines were differentially expressed between lnASCs and obASCs at baseline: complement factor D (1.0-fold in lnASCs; 0.4-fold in obASCs, *P* < 0.001), IL-6 (1.0-fold in lnASCs and 2.1-fold in obASCs, *P* < 0.001), leptin (1.0-fold in lnASCs and 134.9-fold in obASCs, *P* < 0.001), pentraxin-3 (1.0-fold in lnASCs and 0.5-fold in obASCs, *P* < 0.001), CCL5/RANTES (1.0-fold in lnASCs and 1.7-fold in obASCs, *P* < 0.001), and serpineE1 (1.0-fold in lnASCs and 0.6-fold in obASCs, *P* < 0.001). No statistically significant difference was observed in adiponectin, chitinase 3-like 1, IGFBP-3, IL-24, and CCL17/TARC. Following the initial coculture with MCF7 cells, expression of these cytokines and additional cytokines was reduced in lnASCs(c1) compared to obASCs(c1): 0.2-fold and 0.7-fold for chitinase 3-like 1 (*P* < 0.001), 0.3-fold and 1.0-fold for IL-6 (*P* < 0.001), 3.5-fold and 26.2-fold for leptin (*P* < 0.001), 0.4-fold and 0.8-fold for pentraxin-3 (*P* < 0.001), 0.2-fold and 0.6-fold for CCL5 (*P* < 0.001), and 0.6-fold and 1.2-fold for serpinE1 (*P* < 0.001) in lnASCs and obASCs, respectively (Figure 3). Following a second round of coculture, the expression of IL-24 was significantly upregulated in obASCs(c2) to 10.1-fold, whereas lnASCs(c2) expressed 0.8-fold of IL-24 (*P* < 0.001; Figure 3). Levels of chitinase 3-like 1 (2.6-fold in lnASCs(c2) versus 1.4-fold in obASCs(c2), *P* < 0.001), complement factor D (1.8-fold in lnASCs(c2) versus 0.7-fold in obASCs(c2), *P* < 0.001), and pentraxin-3 (0.9-fold in lnASCs(c2) versus 0.6-fold in obASCs(c2), *P* < 0.001) were upregulated in lnASCs(c2) compared to obASCs(c2) (Figure 3). These findings suggest that while levels of induction are important, the temporal expression is equally important. obASCs demonstrated higher levels of these factors following the initial coculture, while lnASCs required two serial cocultures before the expression was increased. Correlating the findings between the secretome and the gene expression profiles, the results indicate that while several key factors are secreted by ASCs, other cytokines are secreted by cancer cells following coculture.

### 3.4. Serial Coculture with obASCs Leads to More Robust Expression of Cytokines in MCF7 Cells, Compared to lnASCs

**Figure 4:**
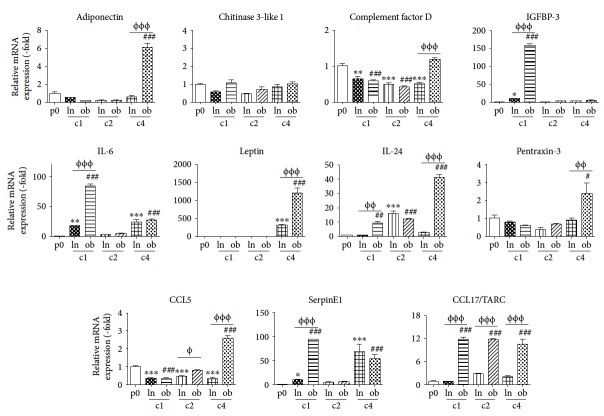
MCF7 cells demonstrated enhanced expression of cytokines following serial coculture. MCF7 cells were passaged or sorted after cocultured with lnASCs or obASCs for 7 days. The gene expression of various cytokines and chemokines in MCF7 cells was assessed by qRT-PCR prior to coculture (naïve) and after one coculture (c1). These lnMCF7(c1) and obMCF7(c1) cells were serially cocultured for another 7, 14, and 21 days to generate lnMCF7(c4) and obMCF7(c4) after four serial cocultures. This lnMCF7(c4) and obMCF7(c4) cells were sorted, and the expression of various factors was assessed by qRT-PCR and normalized to the levels observed in naïve MCF7 cells. Bar: ± SEM. CC: coculture. ^#^*P* < 0.05; ^##^*P* < 0.01; ^###^*P* < 0.001 between naive MCF7 and MCF7s exposed to obASCs. ^Φ^*P* < 0.05; ^ΦΦ^*P* < 0.01; ^ΦΦΦ^*P* < 0.001 between MCF7 cells exposed to lnASCs and MCF7 cells exposed to obASCs. ^∗^*P* < 0.05^∗∗^*P* < 0.01 and ^∗∗∗^*P* < 0.001 between naive MCF7 and MCF7s exposed to lnASCs.

To monitor changes associated with long-term exposure of the cancer cells to lnASC or obASCs, MCF7 cells were serially cocultured with lnASCs or obASCs and sorted. The 11 differentially expressed cytokines identified by the proteome profiler were analyzed further by qRT-PCR to determine whether the MCF7 cells, following serial coculture, were responsible for the enhanced secretion of several cytokines. After the initial coculture with lnASCs or obASCs, MCF7 cells demonstrated enhanced expression of several cytokines, with a more robust response following exposure to obASCs: IGFBP-3 (10.9-fold in lnMCF7(c1) and 158.8-fold in obMCF7(c1), *P* < 0.001); IL-6 (18.2-fold in lnMCF7(c1) and 84.7-fold in obMCF7(c1), *P* < 0.001); serpinE1 (11.2-fold in lnMCF7(c1) and 94.8-fold in obMCF7(c1), *P* < 0.001; Figure 4). Levels of IGFBP-3 and IL-6 reduced to baseline after MCF7 cells were serially cocultured twice (Figure 4). IL-24 expression was higher in lnMCF7(c2) cells (16.0-fold, *P* < 0.001) compared to obMCF7(c2) cells (12.3-fold, *P* < 0.001; Figure 4). Interestingly, long-term serial coculture with obASCs for four cocultures enhanced the expression of the following cytokines, compared to baseline and to MCF7 cells exposed to lnASCs: adiponectin (0.6-fold in lnMCF7(c4) and 6.2-fold in obMCF7 (c4), *P* < 0.001), leptin (316.0-fold in lnMCF7(c4) and 1208.7-fold in obMCF7(c4), *P* < 0.001), IL-24 (2.8-fold in lnMCF7(c4) and 41.1-fold in obMCF7(c4), *P* < 0.001), pentraxin-3 (0.9-fold in lnMCF7(c4); 2.4-fold in obMCF7(c4), *P* < 0.001), and CCL5 (0.3-fold lnMCF7(c4) and 2.6-fold obMCF7 (c4), *P* < 0.001; Figure 4). Other patterns of expression included upregulation of CCL17/TARC in MCF7 cells following one, two, and four serial cocultures with obASCs (11.8-fold in obMCF7(c1) and 11.8-fold in obMCF7(c2) and 10.5-fold in obMCF7(c4); *P* < 0.001). Several genes also demonstrated diminished gene expression levels following one or two serial cocultures, such as complement factor D and CCL5 (Figure 4). These results indicate the temporal effects of serial coculture on gene expression of cytokines. Following exposure to lnASCs, an absent or blunted effect in the gene expression was observed in MCF7s as compared to obASCs.

### 3.5. Serial Coculture with ASCs Alters Cancer Cell Gene Expression, Cell Proliferation, and Invasive Phenotype

**Figure 5:**
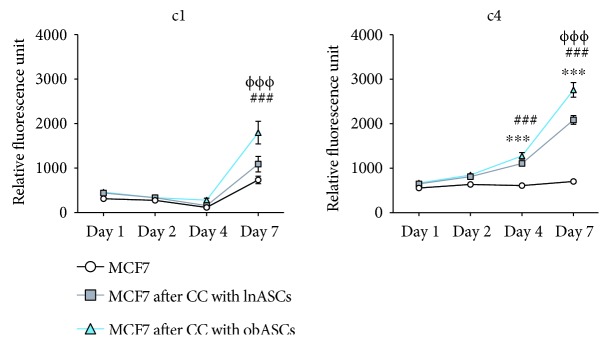
Serial coculture with obASCs enhances proliferation of breast cancer cells. MCF7 cells were serially cocultured with MCF7 cells. Sorted MCF7 cells were assessed by Alamar blue proliferation assay after one coculture (c1) and four cocultures (c4). Bar: ±SEM. CC: coculture. ^∗∗∗^*P* < 0.001 between MCF7 cells and MCF7 exposed to lnASCs. ^###^*P* < 0.001 between MCF7 cells and MCF7 exposed to obASCs. ^ΦΦΦ^*P* < 0.001 between MCF7 cells exposed to lnASCs and MCF7 cells exposed to obASCs.

To assess the proliferative effects of coculture on MCF7 cells, FACS sorted MCF7 cells that were serially cocultured alone, with lnASCs, or with obASCs were evaluated with Alamar Blue. Exposure to lnASCs and obASCs after one coculture enhanced the proliferation of MCF7 cells. lnMCF7(c1) cells (1090 RDU; *P* < 0.001) and obMCF7(c1) cells (1797 RDU, *P* < 0.001) demonstrated enhanced proliferation compared to the MCF7 cells cultured alone (736 RDU), when assessed on day 7 (Figure 5). After four serial cocultures with lnASCs and obASCs, MCF7 cells continued to demonstrate enhanced proliferation (702 RDU in MCF7 and 2085 RDU in lnMCF7(c4) and 2759 RDU in obMCF7(c4), *P* < 0.001). Furthermore, the proliferative effects of long-term coculture of obASCs on MCF7 cells were more robust than the long-term effects of serial coculture with lnASCs over four cocultures (*P* < 0.001; Figure 5).

**Figure 6:**
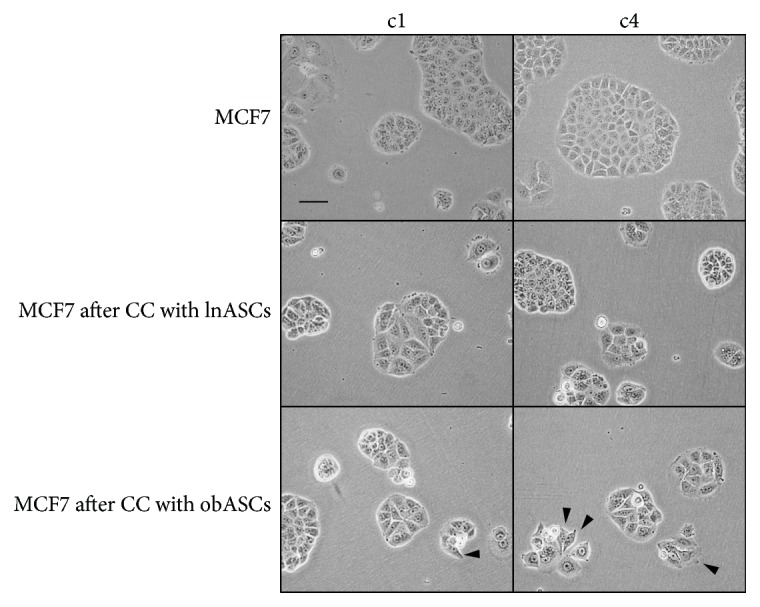
Serial coculture with obASCs enhances invasive phenotype of breast cancer cells. MCF7 cells were serially passaged or serially cocultured with lnASCs or obASCs and assessed morphologically. Arrowheads indicate more mesenchymal-like phenotype. Scale bar represents 200 *μ*m.

To determine whether serial coculture with lnASCs and obASCs altered the cancer cell morphology, cocultured MCF7 cells were sorted and visualized under brightfield microscopy. No significant visible difference was observed in MCF7 cells after the first coculture with lnASCs. The majority of the MCF7 cells cocultured with obASCs maintained the epithelial morphology, while a few sporadic cells displayed more mesenchymal-like phenotype after the initial coculture. After four serial cocultures, MCF7 cells cultured with obASCs displayed even more mesenchymal-like phenotype, with increased invadopodia and increased phenotypical heterogeneity (Figure 6). MCF7 cells cultured alone or serially cocultured with lnASCs maintained a less invasive phenotype (Figure 6). These results suggest that continuous exposure to obASCs enhances cancer cell proliferation and phenotypical heterogeneity, as compared to exposure to lnASCs or cultured alone, indicating that obASCs may play a role in enhancing tumor progression.

## 4. Discussion

Obesity is a significant health issue worldwide, particularly in light of its association with increased risk for a myriad of diseases, including breast cancer. The rapid expansion of fat tissue results in the release of several key cytokines and adipokines involved in chronic inflammation [29]. This local chronic inflammation has the potential to alter cells within the microenvironment of the adipose tissue, including the ASCs. In the current study, lnASCs and obASCs were cocultured with cancer cells to assess their respective effects on the secretome profile of breast cancer cells. Furthermore, the plasticity of ASCs to convert into CAFs and their effects on cancer cells were assessed after long-term serial coculture. Compared to lnASCs cocultured with cancer cells, obASCs cocultured with cancer cells demonstrated a more robust increase in the expression of several key cytokines that have been linked to aggressive breast cancers. Long-term coculture with obASCs resulted in increased proliferation of breast cancer cells. These findings suggest that changes in the local adipose stromal microenvironment in obese subjects impact the biology of ASCs and that these obASCs have an increased capacity to alter breast cancer proliferation and invasion.

To determine whether the increased expression of these key cytokines was associated with an increased rate of conversion into CAFs, lnASCs and obASCs were serially cocultured with MCF7 cells and assessed for the expression of CAF markers. obASCs expressed higher levels of CAF markers after the initial coculture, whereas lnASCs expressed some of the CAF markers only after the second coculture. Several studies have reported that ASCs are a significant source of CAFs and their differentiation into CAFs is driven under the influence of tumor-derived factors [30]. Once converted into CAFs, these cells have been shown to play a central role in regulating inflammation and in promoting proliferation, migration, and angiogenesis of several cancers [31]. The results from our study suggest that obASCs have a higher propensity to convert into CAFs. These are consistent with previous findings that suggest that obASCs, conditioned by their proinflammatory environment, may acquire additional phenotypes that allow for increased migration and invasion [32].

Coculture of lnASCs and obASCs with breast cancer cells resulted in the alteration in the secretome profile. However, to investigate which cell type was responsible for the altered expression levels of cytokines and to assess the effects of long-term coculture, lnASCs and obASCs were assessed at baseline and following serially coculture with breast cancer cells for the cytokines identified by the secretome analysis. Baseline comparisons between lnASCs and obASCs identified higher levels of IL-6, leptin, and CCL5 expression in obASCs compared to lnASCs. These factors all contribute to inflammation and have been shown to enhance tumor growth and metastasis [33–36]. Following continuous exposure to breast cancer cells, the expression of IL-24 in obASCs also continued to increase with each serial coculture, whereas the levels of IL-24 were significantly reduced in lnASCs after the initial coculture. IL-24 has been shown to inhibit the growth of breast cancer cells through the activation of apoptotic pathways and the inhibition of angiogenesis [37, 38]. These results are inconsistent with our current findings and could be associated with differences in human and murine cells. The previously published studies generated breast cancer with the mouse mammary tumor virus and this specie difference could account for the observed discrepancy [37, 38]. Furthermore, these studies validate the effects of IL-24 receptor in triple-negative cell lines [37, 38], rather than the estrogen receptor-positive and progesterone receptor-positive cell line used in the current study, which partially explains the differences observed. Nevertheless, the contrast between our findings and those in previous reports indicates that additional analysis into the role of IL-24 in obASCs through inhibition or knockdown studies would shed light on the effects of IL-24 on breast cancer cells.

Interestingly, breast cancer cells also expressed high levels of cytokines following coculture with lnASCs or obASCs. The gene induction following the initial coculture with obASCs was significantly more robust, particularly in the expression of IL-6, IGFBP-3, and serpinE1. IL-6 has been shown to promote tumor survival, metastasis, and angiogenesis. Human primary mammospheres from node invasive breast cancers express higher levels of IL-6 than from mammospheres from matched nonneoplastic mammary glands [39]. Furthermore, breast cancer cells that were sensitive to drug treatment did not express IL-6, whereas multidrug-resistant breast cancer cells produced high levels of IL-6 [40]. These findings support the data generated from a meta-analysis involving 3224 identified breast cancer patients where IL-6 expression was associated with poor prognosis [41]. Similarly, higher levels of IGFBP-3 have been associated with tumor progression and resistance to treatment due to the intranuclear roles of IGFBPs in transcriptional regulation, induction of apoptosis, and DNA damage repair [42]. Likewise, increased expression of serpinE1 has been correlated with tumor aggressiveness and poor clinical outcomes. The robust expression of IL-6, IGFBP-3, and serpinE1 following coculture with obASCs would suggest that these cancer cells are more aggressive. Furthermore, the enhanced expression of these factors correlated with a more invasive morphology and increased proliferation rate in breast cancer cells following coculture with obASCs compared to lnASCs. Interestingly, the expression of IGFBP-3 and serpinE1 was significantly higher in lnASCs after five serial cocultures, compared to obASCs after five serial cocultures. These findings suggest that lnASCs require significantly more conditioning with breast cancer cells to increase the expression of these factors, whereas obASCs only require the initial exposure to induce similar robust levels of expression. Collectively, these studies suggest that exposure to obASCs results in a more robust induction of cytokines in breast cancer cells and that induction is time dependent, whereby obASCs have the potential to increase the expression of these cytokines following fewer cocultures.

## 5. Conclusion

obASCs increase the proliferation and induce an invasive phenotype in breast cancer cells following long-term serial coculture. Following exposure to breast cancer cells, obASCs express higher levels of CAF markers at earlier time points, indicating that obASCs have a higher propensity to convert into CAFs. Furthermore, analysis of the cytokines and chemokines secreted during coculture demonstrate that breast cancer cells induce higher levels of cytokine expression in obASCs, compared to lnASCs. Moreover, exposure to obASCs results in a more robust level of induction in breast cancer cells. Future studies to inhibit the expression of these factors will shed light on the importance of these factors during breast cancer progression. Identifying the factors relevant to the disease progression will assist in developing targets to reduce the morbidity and mortality associated with breast cancer in obese women.

## Figures and Tables

**Table 1:** Primers for qRT-PCR.

Gene	Forward (5′→ 3′)	Reverse (5′→ 3′)
*Cytokines*		
Adiponectin	AACATGCCCATTCGCTTTAC	AGAGGCTGACCTTCACATCC
Chitinase 3-like 1	TGTGAAGGCGTCTCAAACAG	TACAGAGGAAGCGGTCAAGG
Complement factor D	GGTCACCCAAGCAACAAAGT	CCATGCTGATCTCGAACTCC
IGFBP-3	TAAGTATGGGCAGCCTCTCC	GGTCATGTCCTTGGCAGTCT
IL-6	GTAGCCGCCCCACACAGACAGCC	GCCATCTTTGGAAGGTTC
Leptin	GAAGACCACATCCACACACG	AGCTCAGCCAGACCCATCTA
IL-24	GCCTCTGATTGGTGAATGGT	GGTGTTAAATTGGCGAAAGC
Pentraxin-3	ATTCAGAGGAAGGGCTCACA	TGTTTCATCAAAGCCACCAC
CCL5	CGCTGTCATCCTCATTGCTA	GCACTTGCCACTGGTGTAGA
SerpinE1	CAGACCAAGAGCCTCTCCAC	GACTGTTCCTGTGGGGTTGT
CCL17	CACCCCAGACTCCTGACTGT	CATGGCTCCAGTTCAGACAA

*CAF markers*		
PDGFR*β*	GCACTTTTATCCACCCAGGA	GTACTTGGCTCAGCCTCCAG
NG2	AGTATGGGCATCTCCTGGTG	CATTGACACCCCTAGCCAGT
*α*-SMA	AATGGCTCTGGGCTCTGTAA	TTTGCTCTGTGCTTCGTCAC
VEGF	CGAGGGCCTGGAGTGTGT	CCGCATAATCTGCATGGTGAT
FAP	TACCCAAAGGCTGGAGCTAA	ACAGGACCGAAACATTCTGG
FSP	CAAGTACTCGGGCAAAGAGG	TGCAGGACAGGAAGACACAG

*Housekeeping gene*		
*β*-actin	CACCTTCTACAATGAGCTGC	TCTTCTCGATGCTCGACGGA

CAF = carcinoma-associated fibroblast.
